# Blood Biomarkers in Cardioembolic Stroke

**DOI:** 10.2174/157340310791658767

**Published:** 2010-08

**Authors:** Teresa García-Berrocoso, Israel Fernández-Cadenas, Pilar Delgado, Anna Rosell, Joan Montaner

**Affiliations:** Neurovascular Research Laboratory, Institut de Recerca Vall d’Hebron and Neurovascular Unit Neurology Department. Universitat Autònoma de Barcelona. Medicine Department. Hospital Vall d’Hebron. Barcelona, Spain

**Keywords:** Biomarker, cardioembolic, classification, etiology, natriuretic peptides, stroke.

## Abstract

One promising field in neurovascular diseases investigation is the use of biomarkers to guide stroke etiology diagnosis and classification. Since treatment differs among etiologic subtypes and nowadays many patients receive a diagnosis of undetermined stroke, biomarkers might become an important additional diagnostic tool. In this review we update current knowledge about biomarkers related with cardioembolic stroke etiology (such as BNP and D-dimer proteins, or *PITX2* and *ZFHX3* genes), that in the future, might allow rapidly guiding other diagnostic tests and accelerating the onset of an optimal secondary prevention.

## INTRODUCTION

The use of plasma biomarkers is getting increasingly popular in several fields of medicine. In fact, decision making processes using biomarkers are widely accepted in medical situations such as initiating lipid lowering therapies (by means of low-density lipoprotein plasma levels), diagnosing acute myocardial infarction (with troponins), ruling-out pulmonary embolism suspicions (by D-dimer levels), etc. Interest has also arrived to cerebrovascular diseases, since biomarkers might aid physicians in several steps of stroke evaluation [1].

At present, the diagnosis of stroke remains based on clinical grounds and neuroimaging assessment. As neuroimaging is not a widely available tool, other methods might be useful to early differentiate between hemorrhagic and ischemic stroke or to discard pathologies that simulate cerebral ischemia, mainly in the pre-hospital setting. Biomarkers use might aid in ruling out these “mimics”, such as tumours or epileptic seizures, that account for up to a third of patients with stroke-like symptoms [2], thus avoiding unnecessary urgent transfers, specialist evaluation and extra-testing and improving the cost-efectiveness in stroke field. 

Nowadays intravenous thrombolysis with recombinant tissue-type plasminogen activator (rt-PA) remains the only approved acute treatment for ischemic stroke. However, despite great improvements in the field, still very few stroke patients (1-5%) benefit from these therapies [3]. That small number is due to the delay in hospital admission and diagnosis and the restrictive therapeutic window, since patients must be treated within 4.5 hours after stroke onset [4]. In this area, the applicability of biomarkers could help in improving safety and efficacy of the administration of reperfusion therapies.

Once the diagnosis and hyperacute treatment of ischemic stroke have been done, etiologic classification is critical to discern the best treatment offered both in the stroke units during the acute phase and at the outpatient clinics for the secondary prevention. However, even with a thorough evaluation, the etiology of ischemic stroke remains undetermined in 25-39% of patients [5]. The use of biomarkers for etiologic diagnostic assessment, the focus of this review, might contribute to the reduction of this percentage of cryptogenic strokes and to prescribe the most appropriate secondary treatments (anticoagulant or antiplatelet therapy). 

## STROKE ETIOLOGY CLASSIFICATION SYSTEMS

Given that stroke prognosis, risk of recurrence and choices for management greatly differ between stroke subtypes, several classifications schemes have been developed since the seventies in order to identify the most likely stroke etiology.

In 1993, a new classification system emerged: The TOAST classification [6], which is still the most popular method. According to this system, patients are classified in 11 categories, which are further collapsed into 5 major etiologic groups including cardioembolic (CE) stroke. Interrater reliability was reported by these investigators to be only moderate (kappa=0.54) [7], and therefore, caution was recommended, since disagreements in subtype assignments would remain despite the use of these explicit criteria. Moreover, according to TOAST, stroke of undetermined etiology category would be applied to either patients having two or more definite etiologies or to patients having no cause after a negative extensive work-up, or to patients with incomplete evaluation, leading to great heterogeneity and usually oversizing the group of cryptogenic stroke. 

These are the main reasons why new classifications have been appearing periodically, such as the Lausanne Stroke Registry and the GÉNIC classification [8, 9], which tried to decrease the number of patients classified as having an undetermined etiology, by shifting a great proportion of cases from that group to the atherothrombotic stroke etiology category. 

In 2005, Ay and colleagues [10] raised the question of whether diagnostic advances in stroke evaluation result in more frequent identification of vascular, cardiac and other systemic abnormalities and conflicting results could lead to categorization of most strokes into the undetermined causative category. To avoid the oversizing of the undetermined category, these authors developed an algorithm (SSS-TOAST) that incorporated recent advances in stroke imaging and epidemiology to identify the most probable stroke etiology in the presence of evidence for multiple mechanisms. Based on the weight of evidence, each TOAST sub-type was subdivided into three subcategories: “evident”, “probable” or “possible”. 

The SSS-TOAST assumes that imaging proof of acute infarction is required as a starting point to the accurate classification of ischemic stroke. As example, an otherwise evident or probable mechanism is lowered down to possible if there is no imaging proof of infarction in a location consistent with symptoms. Also, it is required that all patients are evaluated at a minimum level of diagnostic tests, which could be summarized on imaging of the brain (CT/MRI), imaging of extracranial and intracranial vessels (ultrasonography, CT angiography, MR angiography), monitoring the cardiac rhythm, function and structure (electrocardiogram, transthoracic echocardiography) and obtaining relevant blood tests. Further cardiac investigations may be considered relevant and then other cardiac tests (such as transesophageal echocardiography or Holter monitoring) are required.

The same authors [11] developed an automated version of the SSS-TOAST, the Causative Classification System (CCS), which is a web-based, questionnaire-style classification scheme, to facilitate its use in multicenter studies [12] as shown in Fig. (**1**). Finally, it has to be considered that both TOAST classification and SSS-TOAST identify the most likely mechanism of stroke, without taking into account the interaction that might occur when two or more evident mechanisms co-exist.

In summary, although these classifications allow us to have a standard reference language, taken all together, they still have some important limitations. Moreover, these systems are based on associations, because they lack a gold standard, such as pathological confirmation, to define the exact cause of stroke. Thus, we should take them only as the basis of our diagnosis skeleton but we need to develop other ancillary tests to make a correct etiologic classification. Here is where the stroke biomarkers may have an important future role if an etiologic “biological signature” might be studied in accessible body fluids. 

## BIOMARKERS TO IDENTIFY STROKE ETIOLOGIES

At present, only some approaches using biomarkers to evaluate the risk of ischemic stroke appearance are close to be applicable to clinic. That is the case of lipoprotein-associated phospholipase A2 (Lp-PLA2), a circulating enzyme involved in inflammation that is an independent predictor of future stroke among healthy individuals [13]. In fact the FDA has approved recently the blood measurement of Lp-PLA2 to predict the risk of cardiovascular events. Therefore, early stroke detection will permit physicians to prescribe lifestyle changes in order to reduce some risk factors or establish preventive treatments.

Currently there is no single biomarker approved to identify stroke etiology. However several promising candidates have been recently described. In the next part of this review, we will summarize those studies that have tried to differentiate stroke etiologies by using different blood biomarkers. 

The major difficulty in planning a study to identify a biomarker that would help to elucidate ischemic stroke etiology is the absence of a diagnostic standard for ‘stroke of undetermined etiology’, a situation where biomarkers are most needed. For this reason it would be very useful to have a complementary diagnostic tool that could define specifically which etiology is involved in a stroke event in order to provide the optimal treatment. 

The current research is mainly done in blood biomarkers since in a future clinic application the blood tests will be an accessible tool. In the next section, we will review the current knowledge about proteins, mRNA and polymorphisms studied as biomarkers of stroke etiology. Apart from these molecules, other types of biological elements could be indicative of processes related to the stroke pathology and they need to be taken into account, like protein fragments (such as D-dimer), peptides (such as telopeptide from collagen), aminoacids (such as asymmetric dimethylarginine), lipids (such as ceramide) or blood particles (such as platelet-derived microparticles). 

Moreover, we have included also some candidates that although have never been tested in stroke pathology are involved in some diseases considered as risk factors for stroke, or endophenotypes, like atrial fibrillation (AF) for CE stroke. All these promising biomarkers are listed on Table **1**.

## PLASMA PROTEINS RELATED TO STROKE ETIOLOGY

These ideal candidates should be related to the main processes involved in stroke pathophysiology such as hemostasis, inflammation, immune system activation, endothelial damage or oxidative stress.

Abnormalities of coagulation and fibrinolysis may play an important role in the pathogenesis of ischemic stroke. Thus, molecules involved in hemostasis might be useful biomarkers. For example, fibrinopeptide A and prothrombin fragment, that reflect thrombin activity, or D-dimer, a product of fibrin degradation, appear in the circulation when the coagulation system has been activated and red fibrin-rich thrombi have been formed. Those red clots typically originate in diseased cardiac chambers [14], being related to CE stroke etiology [15, 16] (Table **1**). 

In fact, Ageno and colleagues [17] described D-dimer as a marker of CE stroke in a cohort of 126 patients and according to day 1 measurements, the optimal cutoff point for predicting CE stroke was 2.00 microg/mL, resulting in a specificity of 93.2% and in a sensitivity of 59.3%. In this direction and in the largest study ever conducted on biomarkers and stroke etiology, high levels of brain natriuretic peptide (BNP) and D-dimer were independent predictors of CE stroke in 707 ischemic stroke patients [16]. BNP >76 pg/mL OR=2.3 (95%CI, 1.4-3.7, P=0.001); and D-dimer >0.96 microg/mL OR=2.2 (95%CI, 1.4-3.7, P=0.001) were independent predictors of CE stroke and even among patients with transient symptoms (n=155), a high BNP level identified CE etiology (OR= 6.7, 95%CI, 2.4-18.9; P<0.001).

A model combining clinical and biochemical data had a sensitivity of 66.5% and a specificity of 91.3% for predicting cardioembolism. A practical example of the use of those types of models is shown in Fig. (**1B**).

This finding has been recently confirmed by Shibazaki and colleagues [18] who described an optimal cutoff concentration of plasma BNP level to distinguish CE from other stroke subtypes of 140.0 pg/mL, and extended to pro-BNP by Rodríguez-Yáñez and collaborators [19], who studied 262 patients with first ischemic stroke within the first 12 hours, showing that pro-BNP >360 pg/mL was independently associated with CE stroke (OR=28.51, 95% CI, 5.90-136.75, P< 0.0001).

Plasma levels of inflammatory and thrombotic markers in acute ischemic strokes were correlated with the TOAST subtype in a recent study by Licata and colleagues [20], showing how different the pro-inflammatory cytokines profile is between subtypes. In their study, Interleukin (IL)-1beta, IL-6 and Tumor Necrosis Factor (TNF)-alpha were elevated in CE strokes as compared with other etiologies (Table **1**). Otherwise, these cytokines had lower levels in stroke due to small vessel disease (SVD) when compared with all other stroke etiologies. The authors suggested that this might be explained by the different parenchymal zones affected by the infarct and consequently, by the different cellular components involved. Therefore, it seems that the immunological response has a different profile depending on stroke etiology.

This possible association between CE stroke and inflammation is suggested by several studies that are based on the identification of inflammatory serum biomarkers that are elevated in patients with AF. In this population, the successfulness of sinus rhythm maintenance after cardioversion and the risk of CE stroke are related to the inflammatory burden. Indeed, inflammation and thrombosis are intimately related, and an association between AF and TNF-alpha has been demonstrated, moreover, patients with AF have higher levels of IL-6, plasma viscosity and tissue factor (TF), with the later relationship being maintained even after adjustment for confounding factors.

In this line of evidence, one of the most studied proteins is C-reactive protein (CRP), an acute-phase reactant protein that increases in response to different stimuli, like inflammation or infection. It increases within 6 hours and has a peak at about 50 hours after stimulus [21]. CRP has rather been associated with large-artery atherosclerotic (LAA) events, on account of its involvement in the inflammatory process, platelet activation and macrophages differentiation to foam cells [22, 23]. However, Terruzzi and colleagues analyzed recently CRP plasma levels within the 6 first hours after stroke onset and showed higher levels in CE subset than in other etiologies [24]. These and other studies support the role of inflammation on heart diseases like AF [15, 25] (Table **1**). Suggested explanations are that CRP is involved in the coagulation cascade, as CRP binds to phospholipids potencially activated by embolism, or that CRP levels are increased because the common largest extension of brain injury usually seen in CE strokes. 

Nevertheless, some inflammatory markers have been certainly associated with LAA stroke, such as Lipoprotein A [Lp(a)]; that was evaluated in 253 consecutive patients with acute ischemic stroke in whom Lp(a) levels >30 mg/dL were more frequent among the LAA subgroup than among CE (39.4 vs. 11.1%; p < 0.001) [26]. 

Moreover, as there exist also white platelet-rich thrombi, platelet activation is another phenomenon that could provide with some good candidates, like CD40 ligand (CD40L), CD63 or P-selectin mainly associated with LAA strokes although these biomarkers have only been compared among non-CE strokes [27, 28]. However, some investigators have found elevated these markers in AF patients [29, 30], what reveal the necessity of studies that include all stroke etiologies. In fact, very recently, high levels of inflammatory markers shared by both LAA and CE strokes such as CD40 ligand (CD40L) and fetuin-A have been described [31].

A proteomic analysis in serum samples of 24 patients with ischemic stroke (12 LAA and 12 CE patients) identified four spots whose expression intensity was at least four times stronger in LAA patients than in CE patients: haptoglobin (Hp) related protein, serum amyloid A (SAA) (two spots) and Hp alpha chain. In a larger series of patients (n=262) with ischemic stroke using ELISA techniques, Hp levels >1,040 microg/mL identified LAA patients with 95% sensitivity and 88% specificity whereas SAA levels > 160microg/ mL identified LAA patients with 91% sensitivity and 83% specificity [32]. 

The same group also studied 15-deoxy-Delta prostaglandin J2 (15-d-PGJ2) in 552 patients with an acute stroke admitted within 24 hours after symptoms onset [33]. Levels of this prostaglandin were also significantly higher in patients with vascular risk factors (history of hypertension or diabetes) and with LAA infarcts (113.9 pg/mL [95% CI, 81.6 to 139.7]), than in those with SVD (58.7 pg/mL [95%CI, 32.7 to 86.2]), CE (12.1 pg/mL [95%CI, 6.5 to 39.2]), or undetermined origin infarcts (11.4 [5.6 to 24.3] pg/mL); P<0.0001.

The involvement of some pathways in several stroke etiologies reflects the complexity of finding good candidate biomarkers to identify CE strokes. Moreover, the characteristics of the different studies performed (different cohorts, different end-points, different analytical conditions, patients’ treatments…) make hard to reach a consensus about the most suitable biomarkers involved in each etiology. In fact, some etiologies may share mechanisms (and therefore biomarkers) leading to stroke, such as a coronary disease that use to be atherothrombotic but may produce a cardiac discinesia or other heart disfunctions that generate an embolic stroke. The measurement of those markers at different time-points (acute versus subacute or chronic stages of neurovascular disease), includes also some other bias in these studies. 

To overcome all those limitations, more studies involving a large number of ischemic stroke patients to compare biomarkers among etiologies should be done in the next future and it is likely that well-designed diagnostic trials will lead to clinical validation of selected stroke biomarkers. 

## DIFFERENTIAL GENE EXPRESSION AMONG STROKE ETIOLOGIES

Although blood proteins have been the main molecules evaluated as biomarkers, recently some investigations support the usefulness of nucleic acids in the diagnosis of stroke. In 2008, Xu and colleagues presented the first study showing differences of gene expression when they compared CE versus LAA strokes [14]. Using RNA microarrays technology, they found 23 genes that can differentiate both etiologies with a high grade of specificity/sensitivity. Those patients suffering a LAA stroke showed an inflammatory profile, since they had upregulated genes expressed in platelets (such as chemokines: PPBP, PF4, PDGFA and CCR5) and monocytes, both of them involved in the development of the atherosclerotic plaque. On the other hand, genes upregulated in the CE etiology were expressed in neutrophils and modulate the immune response.

Another approach to use nucleic acids as biomarkers is by studying the microRNAs (miRNA), small non-coding RNAs, which control gene expression by both inhibition and activation. In a young Asian cohort, when comparing the expression profile of 836 miRNAs among CE, SVD and LAA strokes using microarrays, 132 miRNAs were useful in predicting etiology [34]. The miRNAs regulated were involved in endothelial/vascular function, angiogenesis, hematopoietic regulation and immune response. 

Even though there are few reports in this field, the promising results obtained by means of these screening techniques using arrays should be analyzed with more detail in the future to find some combination of genes that could characterize each stroke etiology. Furthermore, these studies show how different the scenarios of the stroke pathophysiology are among etiologies and also which processes are altered depending on the cause of stroke. 

## SPECIFIC GENE POLYMORPHISMS OF EACH STROKE ETIOLOGY

At present there are not many studies trying to identify genetic markers of etiology in ischemic stroke. Few analysis have attempted to find these biomarkers but with poor results [35] or with a limited sample size. The great majority of studies have focused in finding genetic risk factors for the different etiologies. In addition, the studies were focused in candidate gene approach, with no validation by other independent studies. 

The most promising results until now are referred to CE stroke and the use of Genome Wide Analysis (GWA) approaches. Several studies using GWAs, that detect over 1 million of single-nucleotide polymorphisms (SNPs) across the genome, have observed significant signals in CE stroke. All these studies have identified SNPs that were associated also with AF, the main risk factor for CE stroke. The DeCODE group found a SNP in the Chromosome 4q25 (rs2200733, near *PITX2* gene) associated with ischemic stroke but only in CE etiology. This gene encodes a catenin-regulated transcription factor associated with AF [36]. In this analysis, the DeCODE group genotyped 1,661 ischemic stroke cases and 10,815 controls using the Infinium HumanHap300 chip that detect over 300,000 SNPs. The most significant SNPs were replicated in two new different cohorts of 2,224 cases and 2,583 controls and in 2,327 cases and 16,760 controls. After the two replications were done, only rs2200733 was statistically associated with CE stroke [37]. Very recently a SNP in *ZFHX3* gene has been associated with AF and with CE stroke [38]. This gene encodes a homeodomain zinc-finger protein that has also been associated with AF. The rs7193343 variant of *ZFHX3* gene was found after the analysis of 2,385 AF cases and 33,752 controls. In a second phase the SNP was genotyped in five ischemic case-control sample sets of European descent comprising 1,036 cases and 3,468 controls and reaching significant association. 

Although there are not specific designed studies developed to find genetic differences between the etiologies of ischemic stroke, the second wave of GWAs in ischemic stroke that will be carried out next year might find these genetic differences through the promising use of endophenotypes. 

## DISCUSSION

Although there are many biomarkers that have been associated with stroke diagnosis, etiology or prognosis, information regarding clinical utility is extremely limited by the scarcity of large, prospective, rigorous, randomized clinical trials. 

Massive sequencing of high quality, full-length cDNA libraries, coupled with proteomics and functional genomic approaches will bring a revolution in biomarkers discovery in the next years. Proteomics seems a promising tool for massive biomarkers identification in the stroke field, mainly directed to diagnosis and treatment response. Also transcriptomics and GWAs studies are recently incorporating new candidate genes that might generate diagnostic and therapeutic targets for specific stroke etiologies. All generated candidates should be carefully validated before they are implemented in clinical routine. 

The ideal biomarker should be very sensitive, specific, reliable, accessible, standardized, cost-effective and easy to interpret. Due to the complex pathophysiology of the stroke, a combined biomarker panel seems more feasible that a single biomarker for a diagnostic test [39]. Including biomarkers from each stroke etiology or combining biochemical markers with bioimaging markers might be an alternative approach [40].

Although point-of-care tools seem ideal in the stroke units setting, information would be useful even if obtained during the first days after stroke onset, allowing the use of standard platforms to get etiological biomarkers results. The use of such biomarkers would allow to drastically reducing the number of undetermined strokes. The identification of CE strokes in cases of paroxistic arrithmias is one of the main indications since this might produce an intensification of the secondary prevention and might be easily missed in routine examinations. For doing this therapeutical decisions we need a very specific test and for guiding other diagnostic tests a very sensitive biomarker would be needed. 

Models of clinical data plus biomarker information and easy interpretation algorithms for clinicians would be mandatory if these markers have to be applied in daily practice. It is likely that future well-designed diagnostic trials will lead to clinical validation of selected stroke biomarkers.

## Figures and Tables

**Fig. (1):**
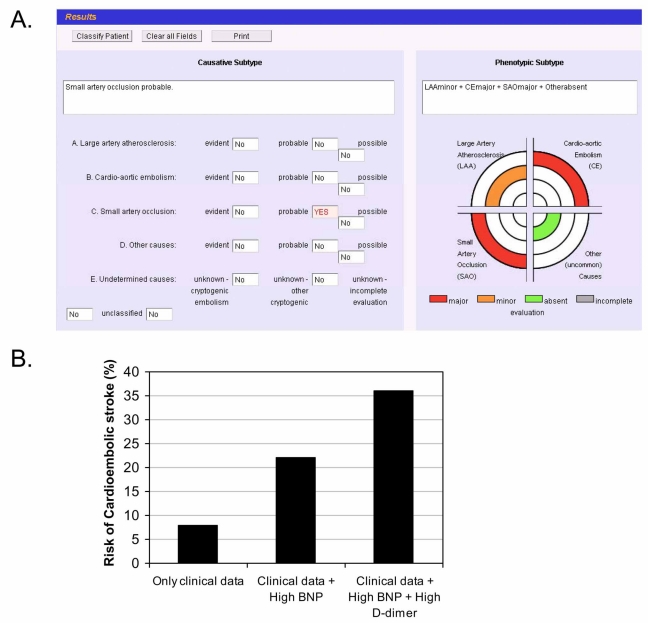
**A.** Visual output from the Causative Classification System (CCS) in a hypothetical case of a 79 years old female patient, with a past history of hypertension, and an acute stroke (NIHSS=15) of undetermined cause using TOAST due to incomplete diagnostic work-up related to lack of all cardiac tests (sinusal rithm on EKG but Holter monitoring and transthoracic echocardiography not performed). **B.** Applying the predictive model described by Montaner and colleagues [16] we increase the risk of cardioembolic stroke from 8% to 36% in the presence of two high cardioembolic stroke related biomarkers (BNP and D-dimer). That would reinforce the necessity of performing all cardiac tests to evaluate a cardioembolic source and the necessity of anticoagulation.

**Table 1:** Candidate Markers of Cardioembolic Stroke, Based on Underlying Pathologies or Endophenotypes

	Name	Accesion #	Reason for Being a Candidate	Ref.
1	Adiponectin	Q15848	Elevated in patients with persistent AF.	[41]
2	Angiopoietin-2 (Ang-2)	O15123	Angiogenic factor. Elevated in AF patients.	[29]
3	Apelin	Q9ULZ1	It seems to be involved in regulation of the angiotensin/vasopressin system. Lower levels in lone AF patients, correlating negatively with proBNP.	[42]
4	Asymmetric dimethylarginine (ADMA)	–	Endogen inhibitor of NOS. ADMA contributes to the thromboembolism in AF.	[43]
5	Atrial natriuretic peptide (ANP)	P01160	Marker of cardiac damage. Higher levels in CE stroke *vs*. other etiologies (262 patients).	[19]
6	Brain Natriuretic peptide (BNP)	P16860	Higher plasma levels in patients suffering from acute CE strokes *vs*. other etiologies. Independent predictor of CE stroke.	[16, 18, 44, 45]
7	Brain natriuretic peptide, proform (pro-BNP)	P16860	Higher levels in CE stroke patients *vs*. other etiologies (262 patients). Pro-BNP might be useful to reclassify undetermined stroke as of CE origin.	[19]
8	Carboxy-terminal telopeptide of collagen type I (CITP)	–	Marker of collagen degradation. Elevated in patients with AF.	[41, 46]
9	CD40 ligand, soluble (sCD40L)	P29965	Inflammatory marker. Elevated in AF patients. No differencies found among etiologies within 107 acute ischemic stroke patients.	[29, 31]
10	CD63	P08962	Platelet activation marker. More positive cells in AF patients (121 patients studied).	[30]
11	Coagulation factor VII (FVII)	P08709	A promoter polymorphism is associated with risk reduction of CE stroke in AF patients.	[47]
12	C-reactive protein (CRP)	P02741	Risk marker for AF. Increased in acute CE stroke respect other etiologies (648 patients studied).	[15, 24]
13	D-dimer	–	Breakdown product of fibrin, elevated in patients with AF. Independent predictor of CE stroke (707 ischemic stroke patients).	[15-17]
14	E-selectin, soluble	P16581	Marker of endothelial activation. Elevated in AF patients.	[48]
15	Fibrinopeptide A	P02671	Increased in patients with AF. It reflects thrombin activity.	[15]
16	Interleukin-1beta (IL-1beta)	P01584	Higher levels in subacute CE stroke *vs.* other etiologies (120 patients involved).	[20]
17	Interleukin-6 (IL-6)	P05231	Pro-inflammatory cytokine. Elevated in AF patients and in subacute CE stroke *vs.* other etiologies (120 patients studied).	[15, 20, 25]
18	Matrix metalloproteinase-1 (MMP-1)	P03956	Patients with AF had MMP-1 reduced.	[49]
19	Matrix metalloproteinase-9 (MMP-9)	P14780	Elevated in AF patients *vs*. controls.	[50]
20	Neuropeptide Y (NPY)	P01303	Elevated in AF patients *vs*. controls.	[50]
21	Paired-like homeodomain (PITX2)	Q99697	Transcription factor associated with AF. Gene polymorphism associated with CE stroke.	[36, 37]
22	Platelet factor 4 (PF-4)	P02776	Marker of platelet activation in AF patients.	[15]
23	Platelet microparticles (PMP)	–	Higher number of particles in AF than in healthy controls.	[51]
24	Prothrombin fragment 1.2 (F1+2)	–	Marker of thrombogenesis. Increased in AF patients.	[49]
25	P-selectin (CD62P)	P16109	More positive cells in AF patients with high risk of stroke.	[30]
26	p-Selectin, soluble	P16109	Marker of platelet activation in AF patients.	[15, 30]
27	Tissue factor (TF)	P13726	Coagulation. Higher levels in AF patients.	[15]
28	Tissue inhibitor of metalloproteinases-1 (TIMP-1)	P01033	Increased in AF patients.	[49]
29	Transforming growth factor-beta (TGF-beta)	P01137	Profibrotic cytokine. Higher levels predict persistent AF.	[52]
30	Tumor necrosis factor-alpha (TNF-alpha)	P01375	Pro-inflammatory cytokine. Elevated in AF patients and in subacute CE stroke *vs.* other etiologies (120 patients studied).	[15, 20]
31	Vascular endothelial growth factor (VEGF)	P15692	Elevated in AF patients	[29]
32	von Willebrand factor (vWF)	P04275	Marker of endothelial damage/dysfunction. Higher plasma levels in AF patients, where predicts cardiovascular events.	[15, 53]
33	Zinc finger homeobox 3 (ZFHX3)	Q15911	Gene polymorphism associated with AF and CE stroke.	[38]

## ABBREVIATIONS

15-d-PGJ2: = 15-deoxy-Delta protaglandin J2
AF: = Atrial fibrillation
BNP: = Brain natriuretic peptide
CCR5: = C-C motif chemokine 5
CCS: = Causative classification system
CD40L: = CD40 ligand
CE: = Cardioembolism
CRP: = C-reactive protein
CT: = Computed tomography
FDA: = Food and Drug Administration
GÉNIC: = Étude du profil génétique de l’Infarctus Cérébral
GWA: = Genome wide analysis
Hp: = Haptoglobin
IL-1beta: = Interleukin-1beta
IL-6: = Interleukin-6
LAA: = Large-artery atherosclerosis
Lp(a): = Lipoprotein A
Lp-PLA2: = Lipoprotein-associated phospholipase A2
MRI: = Magnetic resonance imaging
PDGFA: = Platelet-derived growth factor alpha
PF4: = Platelet factor 4
PITX2: = Paired-like homeodomain
PPBP: = Pro-platelet basic protein
rt-PA: = Recombinant tissue-type plasminogen activator
SAA: = Serum amyloid A protein
SNP: = Single-nucleotide polymorphism
SVD: = Small vessel disease
TF: = Tissue factor
TNF-alpha: = Tumor necrosis factor-alpha
TOAST: = Trial of Org 10172 in Acute Stroke Treatment
ZFHX3: = Zinc finger homeobox 3
